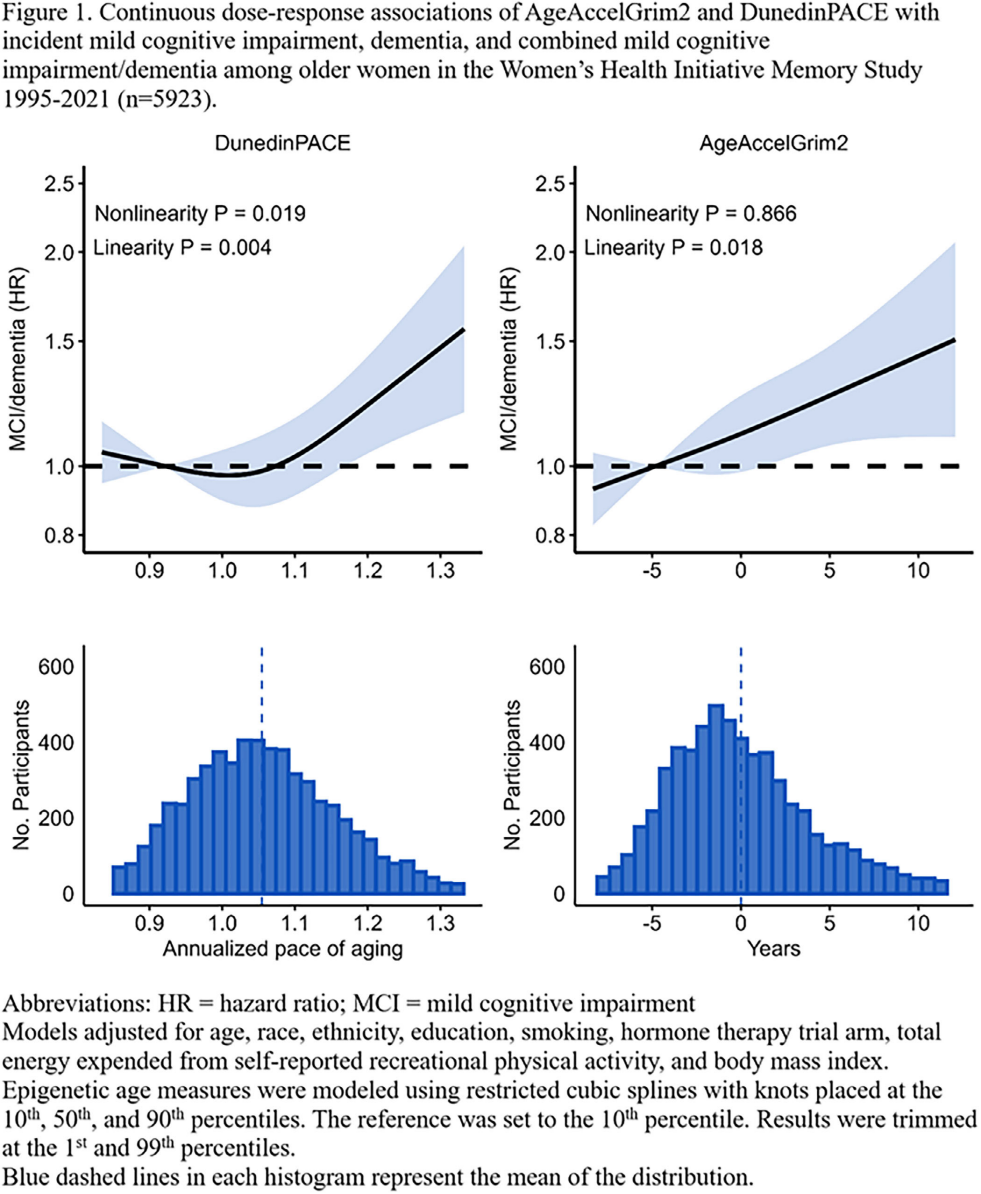# Prospective associations of epigenetic age estimators with incident mild cognitive impairment and dementia among older women: The Women's Health Initiative Memory Study

**DOI:** 10.1002/alz70856_098954

**Published:** 2025-12-24

**Authors:** Steve Nguyen, Ake Lu, Steve Horvath, Mark A. Espeland, Stephen R. Rapp, Adam Maihofer, Caroline Nievergelt, Andrea Z. LaCroix, Linda K. McEvoy, Susan M. Resnick, Kenneth Beckman, Aladdin H. Shadyab

**Affiliations:** ^1^ University of California San Diego, La Jolla, CA, USA; ^2^ Altos Labs, Cambridge, Cambridgeshire, United Kingdom; ^3^ Wake Forest University, Winston‐Salem, NC, USA; ^4^ Kaiser Permanente Washington Health Research Institute, Seattle, WA, USA; ^5^ National Institute on Aging, National Institutes of Health, Baltimore, MD, USA; ^6^ University of Minnesota Genomics Center, Minneapolis, MN, USA

## Abstract

**Background:**

Multiple epigenetic age estimators, indicating biological aging relative to chronological age, have been developed and shown to associate with health outcomes including cardiovascular disease (CVD) and mortality. Relatively few large studies have examined the prospective associations of epigenetic age estimators with rigorously adjudicated mild cognitive impairment (MCI) or dementia.

**Methods:**

Women enrolled in the Women's Health Initiative Memory Study (*n* = 5923, mean age=70.0±3.8 years) without MCI or dementia at baseline (1995‐1998) underwent annual cognitive testing. MCI and dementia were centrally adjudicated by a multidisciplinary panel of experts. We calculated the following epigenetic age estimators: Horvath age acceleration (AA), Hannum AA, AgeAccelPheno; and their corresponding principal component versions, Intrinsic epigenetic AA, extrinsic epigenetic AA, AgeAccelGrim2, and DunedinPACE. Multivariable Cox proportional hazards models estimated hazard ratios (HR) and 95% confidence intervals (CI) for epigenetic age estimators with incident MCI or dementia, adjusting for age, education, race/ethnicity, smoking, hormone therapy trial arm, physical activity, and body mass index (BMI). We additionally evaluated effect modification by age, BMI, physical activity, *APOE ε4* carrier status, and race/ethnicity.

**Results:**

There were 1267 incident MCI/dementia events (780 MCI and 758 dementia events) over a median follow‐up of 9.3 years. Adjusted HR (95% CI) for DunedinPACE quartiles were 1.00 (reference), 1.00 (0.86‐1.17), 0.89 (0.76‐1.05), and 1.22 (1.03‐1.44; *p*‐trend=0.02). Adjusted HR (95% CI) for AgeAccelGrim2 quartiles were 1.00 (reference), 1.09 (0.93‐1.27), 1.25 (1.07‐1.47), 1.21 (1.01‐1.46; *p*‐trend=0.01). Restricted cubic spline analysis suggested a non‐linear association for DunedinPACE(*p*‐nonlinear=0.02) but not for AgeAccelGrim2 (*p*‐nonlinear=0.87; Figure 1). Associations were consistent in direction and magnitude across *APOE ε4* carrier status for DunedinPACE and AgeAccelGrim2. The other epigenetic age estimators were not associated with incident MCI/dementia.

**Conclusions:**

In one of the largest and most comprehensive studies of epigenetic clocks and cognitive outcomes to date, we found that faster biological aging as indicated by two epigenetic age‐acceleration estimators––developed to predict mortality (AgeAccelGrim2) and capture multiple organ system functioning (DunedinPACE)––also predicted incident MCI/dementia. These results show that accelerated epigenetic age based on these estimators could help identify older women at risk for MCI/dementia, and provide insight into pathophysiological mechanisms.